# Retrospective European Multicentric Evaluation of Selective Transarterial Chemoembolisation with and without Balloon-Occlusion in Patients with Hepatocellular Carcinoma: A Propensity Score Matched Analysis

**DOI:** 10.1007/s00270-021-02805-5

**Published:** 2021-03-11

**Authors:** Rita Golfieri, Mario Bezzi, Gontran Verset, Fabio Fucilli, Cristina Mosconi, Alberta Cappelli, Alexandro Paccapelo, Pierleone Lucatelli, Nicolas Magand, Agnes Rode, Thierry De Baere

**Affiliations:** 1 Department of Radiology, IRCCS Azienda Ospedaliero-Universitaria Di Bologna, via Albertoni 15, 40138 Bologna, Italy; 2 Università Degli Studi Di Bologna, Bologna, Italy; 3 Vascular and Interventional Radiology Unit, Department of Diagnostic Service, Sapienza University of Rome, Rome, Italy; 4 Department of Gastroenterology, Hepatopancreatology and Digestive Oncology, Erasme Hospital, Université Libre de Bruxelles (ULB), Brussels, Belgium; 5 Radiology Unit, S. De Bellis National Institute of Gastroenterology Research Hospital, Castellana Grotte (BARI), Bari, Italy; 6 Diagnostic and Interventional Radiology Department, Croix Rousse Hospital, Hospices Civils de, Lyon, France; 7 Department of Interventional Radiology, Gustave Roussy Cancer Center, Villejuif, France

**Keywords:** Hepatocellular carcinoma, Balloon-occluded transcatheter arterial chemoembolization, Treatment effect, Transcatheter arterial chemoembolization, Prognosis, Balloon-occluded arterial stump pressure, Micro-balloon catheter

## Abstract

**Purpose:**

The aim of this retrospective multicentric study was to compare the tumour response rates of Balloon-occluded Transarterial Chemoembolisation (B-TACE) to non-B-TACE using propensity score matching (PSM) in patients with hepatocellular carcinoma and to investigate the clinical benefit, such as lower rates of TACE re-intervention achieved using B-TACE.

**Material and Methods:**

The B-TACE procedures (*n* = 96 patients) were compared with a control group of non-B-TACE treatments (*n* = 434 pts), performed with conventional (cTACE) or drug-eluting microspheres TACE (DEM-TACE). Data were collected from six European centres from 2015 to 2019.

Objective responses (OR) and complete response (CR) rates after the first session and the number of TACE re-interventions were evaluated using PSM (91 patients per arm).

**Results:**

The best target OR after PSM were similar for both B-TACE and non-B-TACE (90.1% and 86.8%, *p* = 0.644); however, CR at 1–6 months was significantly higher for B-TACE (59.3% vs. 41.8%, *p* = 0.026). Patients treated with B-TACE had a significantly lower retreatment rate during the first 6 months (9.9%% vs. 22.0%, *p* = 0.041). Post-embolisation syndrome (PES) rates were 8.8% in non-B-TACE and 41.8% in B-TACE (*p* < 0.001), with no significant differences between groups regarding major adverse events.

**Conclusion:**

B-TACE is safe and effective, achieving higher CR rates than non-B-TACE. Patients undergoing B-TACE had a significantly lower retreatment rate within the first 6 months but higher PES rates.

**Level of Evidence III:**

Level 3, retrospective study.

**Supplementary Information:**

The online version contains supplementary material available at 10.1007/s00270-021-02805-5.

## Introduction

Transarterial chemoembolisation (TACE) can be performed using two different TACE techniques: conventional TACE (cTACE), which uses Lipiodol®, and TACE with drug-eluting microspheres loaded with cytotoxic agents (DEM-TACE), without significant differences in either tumour response or overall survival [1–3]. Based on recent guidelines [4–6], either technique can be utilised, with the choice left to the physician. Selective or superselective treatment is strongly recommended in the current international guidelines [4, 7–15] in order to maximise tumour necrosis as it is thought that, by also filling the portal venules within the nodule periphery, better antitumoural effects are obtained [7, 8].

Liver arterial hemodynamics [16] involved two types of the terminal hepatic artery; the first ends within the portal tract through the peribiliary vascular plexus (PBP). This is the drainage area of intrahepatic metastasis and microsatellitosis (and therefore of the residual /relapse of the disease) which is often not reached by the chemotherapy mixture if injected in free flow during cTACE/DEM-TACE [17, 18]. This area should be included in the treatment, constituting some “safety margin” at the periphery of the target tumour, such as in surgery, radiofrequency ablation (RFA) and TACE. The second terminal artery, called the “isolated artery”, penetrates the liver parenchyma unaccompanied by the bile duct, creating extrahepatic and intersegmental collaterals after repeated TACE procedures, which can feed a residual tumour.

In fact, a significant limitation of all TACE treatments is the high rate of tumour recurrence and refractoriness, frequently encountered after repeated cTACE, with the 5-year tumour recurrence rate reaching 70% [19]. Patients who show a complete response (CR) to initial TACE achieve significantly longer overall survival (OS) [20], suggesting the importance of achieving a CR in the first treatment as the overall response rate decreases with additional TACE sessions as compared with the response to the first TACE [21, 22].

In order to increase the CR rate, in 2009, Balloon-occluded TACE (B-TACE) was introduced by Irie et al., being performed with a balloon microcatheter inflated within the tumour-feeding arteries during selective cTACE [23, 24]. By modifying the flow, it allowed for an increase in tumour coverage by the drug while preventing the backflow of embolic material proximally. In B-TACE, by adding arterial blockage, embolisation of both the hepatic artery and the portal vein can be achieved, leading not only to necrosis of the tumour core, but also to peritumoural infarction, including microsatellites, providing a “transcatheter subsegmentectomy” responsible for atrophy of the surrounding liver parenchyma. During TACE, Lipiodol-emulsion or loaded microspheres are forced into tumour vascularisation by the arterial blood pressure; they flow into the cancer nodules but are often washed out to the portal venous system by means of arterioportal communication [25]. Owing to the blocking effect of the proximal arterial flow, B-TACE improves the uptake of the embolic agent into the cancer nodules with denser deposition within the lesion, and includes the peritumoural area, in cases in which the balloon-occluded arterial stump pressure (BOASP) is 64 mmHg or less, as has been reported, and in the absence of large collateral arteries [23, 24].

Some previous reports have compared the results of safety and efficacy on patients treated with B-TACE versus non-B-TACE; all were single centre cohort studies involving a relatively small number of subjects and the majority of them used miriplatin. Their results, however, demonstrated that the therapeutic effect of B-TACE was better than that of non-B-TACE [26–29]. Therefore, an investigation regarding the type of response achieved in a larger number of patients in a larger number of institutions was needed in order to evaluate safety, response rates and clinical benefits. Since the complete response rate is unsatisfactory after TACE and local tumour regrowth is common, indicating the need for retreatment within 1 to 6 months, the contribution of B-TACE to this point of weakness of TACE was also specifically investigated.

The present multicentric study was a retrospective comparative evaluation of B-TACE versus non-B-TACE treatments, carried out using propensity score matching (PSM), with the aim of first evaluating the efficacy of B-TACE in terms of objective (OR) and complete response (CR) rates according to the modified Response Evaluation Criteria in Solid Tumors (mRECIST) criteria after one session and, second, investigating whether a clinical benefit, such as lower rates of TACE re-intervention (performed according to an on-demand treatment strategy), could be achieved using B-TACE.

### Materials and Methods

The study was carried out retrospectively after approval by the Institutional Ethics Committee and in compliance with the Declaration of Helsinki. Written informed consent was obtained from all patients.

### Patient Population

This retrospective study included a population, collected from 6 European centres, of 530 patients affected by early or intermediate stage HCC not amenable to curative treatment, who were treated between January 2015 and December 2019 with either B-TACE or non-B-TACE performed during the current clinical practice. Ninety-six of the 530 patients were treated with B-TACE with either Lipiodol-based cTACE or DEM-TACE (Table 1-Supplementary material, Fig. 1), and 434 patients were treated with non-B-TACE (234 with cTACE and 200 with DEM-TACE). The demographic distribution and patient characteristics of the B-TACE and the non-B-TACE populations are shown in Table 1, and the baseline characteristics of the B-TACE patients, treated with either cTACE or DEM-TACE, are shown in Table 2-Supplementary material.

**Fig. 1:**
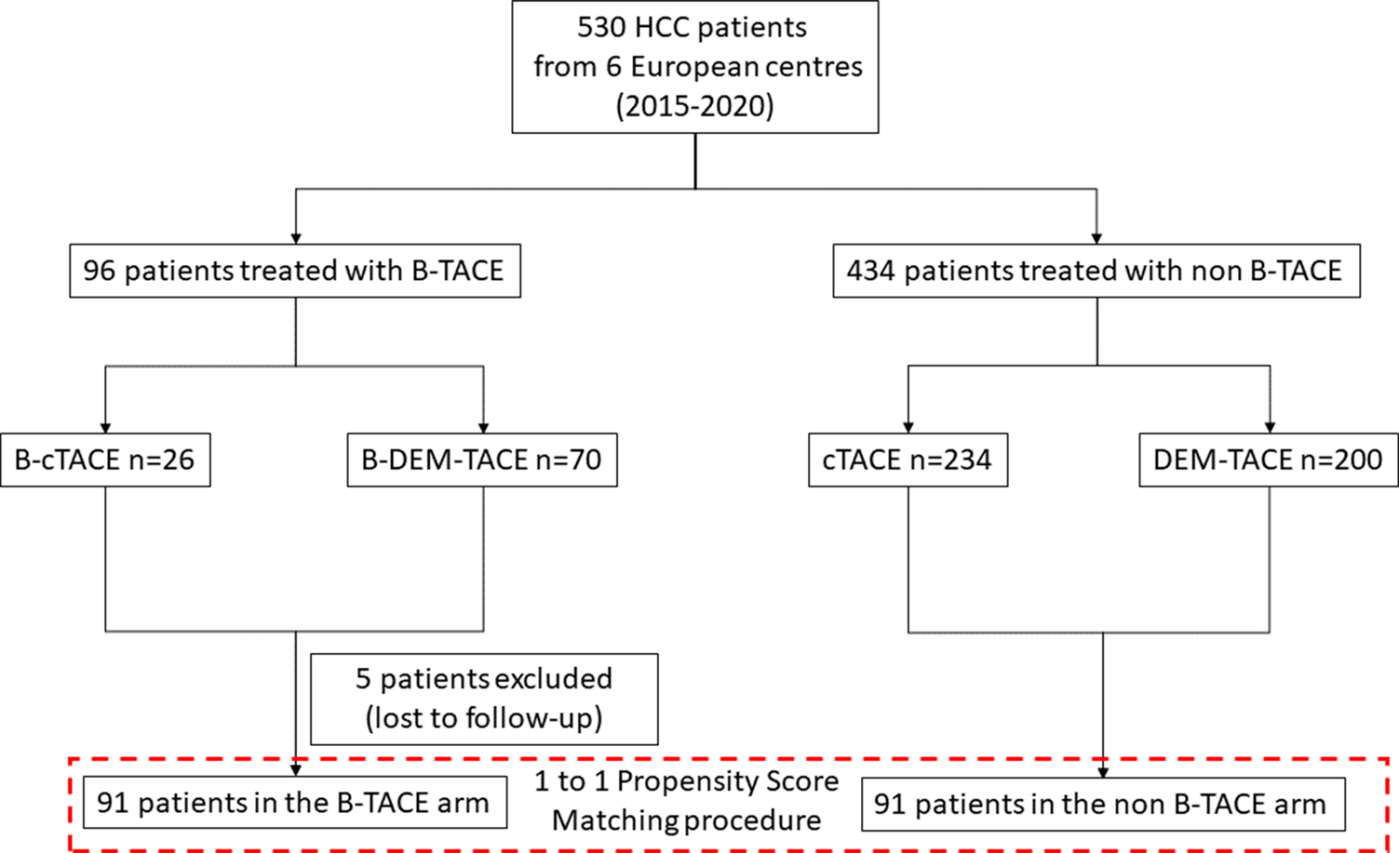
Study profile

**Table 1:** Study population including all patients enrolled for a comparison of the number of nodules, age, gender, type of TACE and child–pugh class, before and after propensity score matching (PSM)

Before PSM	Total no. of patients (*n* = 530)		Non-B-TACE (*n* = 434)		B-TACE (*n* = 96)		*P*
*Gender*							
Male	352	(66.4%)	276	(63.6%)	76	(79.2%)	0.004^A^
Female	178	(33.6%)	158	(36.4%)	20	(20.8%)	
*Age, mean (range)*	66.10	(27–91)	65.44	(27–87)	69.08	(40–91)	0.002^B^
*Type of TACE*							
DEM-TACE	272	(51.3%)	200	(46.1%)	72	(75.0%)	< 0.001^A^
cTACE	258	(48.7%)	234	(53.9%)	24	(25.0%)	
No. nodules, mean (range)	2.19	(1–10)	2.24	(1–10)	1.96	(1–9)	0.120^B^
*Child–Pugh at first TACE*							
A	359	(75.1%)	288	(66.4%)	71	(74.0%)	
B	111	(23.2%)	86	(19.8%)	25	(26.0%)	0.858^C^
C*	8	(1.7%)	8	(1.8%)	0	(0.0%)	

After PSM	Total no. of patients (*n* = 182)		Non-B-TACE (*n* = 91)		B-TACE (*n* = 91)		*P*
*Gender*							
Male	155	(85.2%)	80	(87.9%)	75	(82.4%)	0.405^A^
Female	27	(14.8%)	11	(12.1%)	16	(17.6%)	
Age, mean (range)	68.11	(40–91)	67.62	(47–87)	68.59	(40–91)	0.553^B^
*Type of TACE*							
DEM-TACE	126	(69.2%)	57	(62.6%)	69	(75.8%)	0.077^A^
cTACE	56	(30.8%)	34	(37.4%)	22	(24.2%)	
No. of nodules, mean (range)	2.02	(1–9)	2.08	(1–7)	1.97	(1–9)	0.624^B^
*Child–Pugh at first TACE*							
A	139	(76.4%)	72	(79.1%)	67	(73.6%)	
B	41	(22.5%)	17	(18.7%)	24	(26.4%)	0.437^C^
C*	2	(1.1%)	2	(2.2%)	0	(0.0%)	

^A^Fisher's Exact test; ^B^Student’s t test; ^C^Mann–Whitney U test

^*^Treated with superselective non B-TACE although classified in child–pugh C class, due to small amount of ascites corrected by diuretics

The inclusion criteria were a Child–Pugh class of up to B8 (Patients classified in Child–Pugh C class were also included, provided that this was due to small amount of ascites corrected by diuretics), a Barcelona Clinic Liver Cancer (BCLC) stage of up to B, and large and/or multinodular tumours not eligible for resection or ablation. The exclusion criteria were BCLC stage C, portal vein thrombosis (complete or partial obstruction of the portal vein, due to the presence of a chronic, acute or neoplastic thrombus in the vasal lumen), extrahepatic metastasis, previous systemic treatment, a platelet count < 50,000 and a bilirubin level > 3 mg/dL.

All treated lesions had previously been untreated. The patient database reported in this manuscript has never been published before.

The B-TACE population included 96 patients (179 nodules). Five patients were excluded since they were lost to follow-up (Fig. 1). In the B-TACE population, 22 patients were treated with cTACE (B-cTACE) and 69 with DEM-TACE (B-DEM-TACE). In the B-TACE patients, the mean BOASP was measured for the majority (65/96) of the patients and a comparative analysis between BOASP values and response to B-TACE was performed.

The response after B-TACE and the number of re-treatments required were then compared with those obtained in the control group (*N* = 434) of non-B-TACE patients who underwent superselective cTACE and DEM-TACE performed using only a standard microcatheter without the support of balloon-occlusion; the latter were registered on the institutional database and selected as having similar characteristics (demographic and disease).

### TACE and B-TACE Technical Procedures

*-cTACE* was carried out using selective or superselective catheterisation of the hepatic arteries feeding the lesions using a coaxial microcatheter (2.7–2.8 Fr) inserted as peripherally as possible. A mixture of epirubicin (Farmorubicin®; Pfizer, Latina, Italy) and iodised oil (Lipiodol®; Guerbet, Milan, Italy) was injected under fluoroscopy, followed by embolisation using Spongel (Gelitaspongel®) particles until complete blockage of the tumour-feeding vessels was demonstrated. The mean dose of epirubicin for cTACE was 40.5 mg (range, 20–75 mg), and the mean dose administered of Lipiodol® per treatment was 8.0 mL (range, 4–15 mL).

In the B-cTACE treatments, a mean dose of 8.3 ml of Lipiodol® was injected (range 4–15 ml) mixed with a mean epirubicin dose of 46.1 mg (range 17–100 mg). Epirubicin doses were similar between cTACE and B-cTACE (*p* = 0.401).

*- DEM-TACE* was carried out using drug-eluting microspheres (Life Pearl®, Terumo Europe NV, Leuven, Belgium [100 ± 25 µm and 200 ± 50 µm]) pre-loaded with 50 mg doxorubicin per syringe (2 syringes). One hundred and six patients (39.2%) were treated with 100 µm particles, 45 patients (16.6%) were treated with 200 µm particles and 119 patients (44.1%) were treated with both particles, starting with the smaller particles followed by the larger particles. The mean dose administered per treatment for DEM-TACE was 74 ± 17 mg (range 25–150 mg) of doxorubicin.

In the B-DEM-TACE patients Life Pearls 100 µm with a doxorubicin mean dose of 33.5 mg and Life Pearls 200 µm loaded with a doxorubicin mean dose of 23.5 mg were injected. The mean total dose of doxorubicin was 59.3 mg (range 12.5–100). Doxorubicin doses were significantly higher in DEM-TACE than in B-DEM-TACE (*P* = 0.001). Among the two groups of B-TACE (B-cTACE and B-DEM-TACE), a trend for differences was observed in the dose of drugs, favouring higher dosages in the B-DEM-TACE arm (*p* = 0.054), consistent however with lesion size (*p* < 0.001).

**Fig. 2:**
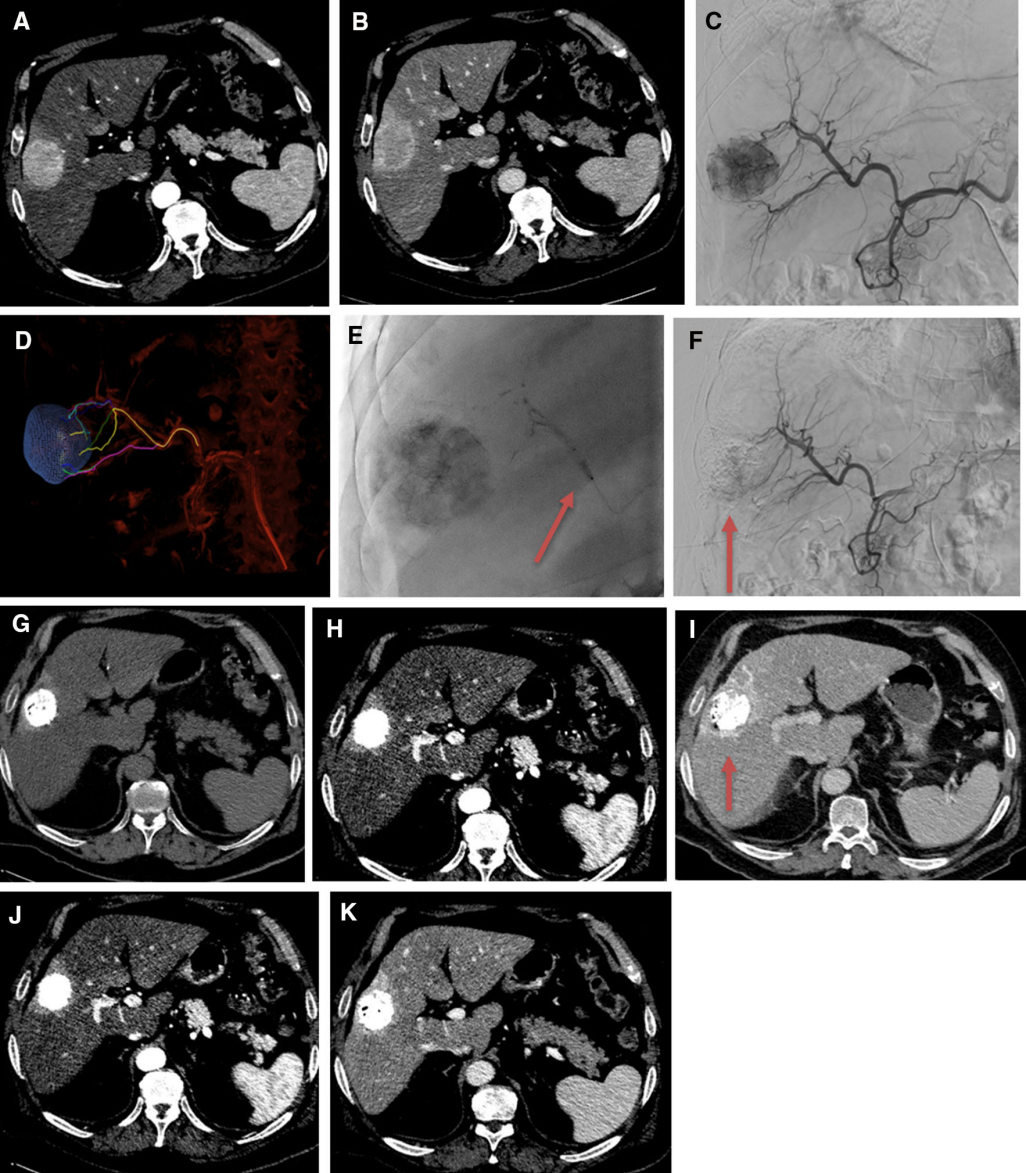
**A**, **B** pretreatment computed tomography (CT): a 50 mm HHC is seen in segment V, highly hypervascular in the arterial phase **A** with a corona enhancement in the portal phase **B**; **C**, **D** pretreatment angiogram and cone beam CT preliminary to balloon inflation and balloon-occluded arterial stump pressure (BOASP) measurement; **E**, **F** B-TACE treatment with lipiodol-epirubicin injection during balloon inflation immediately after BOASP measurement (arrow) and final angiographic control **F**; **G**, **H**, **I** Follow-up CT at 1 month [pre-contrast **G**, arterial **H** and venous phases **I**], showing a size reduction to 43 mm and the dense distribution of Lipiodol, also including the drainage area; **J**, **K** Follow-up CT at 6 months: an additional decrease in size to 35 mm, no viable tumour

B-TACE was performed using a balloon microcatheter (Occlusafe®, Terumo Europe NV, Leuven, Belgium), that is a 2.8 Fr microcatheter with an occlusion balloon on the tip. The micro-balloon is made of compliant polyurethane and is 10 mm in length. The diameter ranges from 1 to 4 mm, according to the volume injected. The balloon microcatheter works on a 0.014′’ platform. Micro-balloon inflation was carried out using a solution of 1:4 of contrast media/saline. Once the balloon microcatheter was positioned, the arterial pressure at the tip of the microcatheter was measured, using an invasive arterial pressure measurement kit. Subsequently, the balloon was inflated to occlude the flow and obtain a reduction in the BOASP; the BOASP was measured before and after inflation of the balloon. The embolisation was then performed, according to routine clinical practice as described above (Fig. 2). The endpoint of the embolisation was target lesion tumour staining, with opacification of the portal venous radicles or opacification of the arterio-arterial anastomoses.

### Assessment of the Tumour Radiological Response and Follow-Up

Patients underwent imaging assessment (quadriphasic computed tomography [CT] or dynamic magnetic resonance imaging [MRI]) at 1, 3 and 6 months after TACE in order to evaluate the best target radiological response, defined as the best response recorded during the first 6 months of follow-up evaluations according to the modified Response Evaluation Criteria in Solid Tumors (mRECIST) [30]. Images were evaluated by the investigator/radiologist at each site.

The primary outcome measured both the CR and the partial response (PR) rates. The OR rate was considered to be the percentage of patients with a CR and a PR of the target lesion(s) (maximum 2 lesions) obtained during the 6-month follow-up period. When viable nodules were detected on follow-up, on-demand TACE was performed if the liver function was Child–Pugh A/B, and portal venous thrombus was not seen in the lobar branch or main trunk.

Early retreatments were considered to be the number of repeated procedures performed within 6 months due to a residual/recurrent tumour.

### Study Safety Outcomes

The study recorded as per procedure, the incidence of biological and clinical adverse events (AEs) according to the CIRSE classification system and Common Terminology Criteria of Adverse Events (CTCAE) version 5.0 [31, 32] for serious adverse events occurring within 30 days post-procedure, based on information collected regarding the event. The radiological safety evaluation carried out at the 1-month follow-up included the detection of liver bile duct injuries, such as segmental dilation or biloma formation, liver infarction in the non-tumoural parenchyma and the appearance of indirect imaging features of vascular damage. Post-embolisation syndrome (PES) was defined as the onset of fever, nausea/vomiting and pain, and was clinically evaluated during the patient’s hospital stay.

### Statistical Analysis

Data are presented as means, ranges and frequencies. The Chi-squared, Fisher’s exact, Student’s t and Mann–Whitney U tests were used. Patients were matched in a Propensity Score Matching (PSM) procedure by a one-to-one ratio for age, gender, number of nodules, Child–Pugh score, and type of TACE (conventional or DEM-based) with a match tolerance of 0.2, giving priority to exact matches. The baseline characteristics were analysed before and after the PSM.

All the tests were two-tailed; a *P*-value < 0.05 was considered statistically significant. All the statistical analyses were carried out using IBM SPSS 25.0 (SPSS Inc., Armonk, NY, USA).

### Results

The best target responses were similar between the two treatments, with ORs of 88.9 and 90.1, and CRs of 50.2% and 59.3% for TACE and B-TACE, respectively (Table 2). After PSM, a slightly better OR was observed for B-TACE (90.1% vs. 86.8% *p* = 0.644), albeit not significant; however, the CR was significantly higher for B-TACE (59.3% vs. 0.41.8%, *p* = 0.026).

**Table 2:** Best target responses before and after propensity score matching (PSM) for number of nodules, age, gender, type of TACE and child–pugh class

Before PSM	Total no. of patients (*n* = 525)		Non-B-TACE (*n* = 434)		B-TACE (*n* = 91)		P
Best target response							
Complete response	272	(51.8%)	218	(50.2%)	54	(59.3%)	0.134^A^
Partial response	196	(37.3%)	168	(38.7%)	28	(30.8%)	
Stable disease	33	(6.3%)	28	(6.5%)	5	(5.5%)	
Progressive disease	24	(4.6%)	20	(4.6%)	4	(4.4%)	
OR: complete + partial response	468	(89.1%)	386	(88.9%)	82	(90.1%)	0.854^B^

After PSM	Total no. of patients (*n* = 182)		Non-B-TACE (*n* = 91)		B-TACE (*n* = 91)		*P*
Best target response							
Complete response	92	(50.5%)	38	(41.8%)	54	(59.3%)	0.026^A^
Partial response	69	(37.9%)	41	(45.1%)	28	(30.8%)	
Stable disease	14	(7.7%)	9	(9.9%)	5	(5.5%)	
Progressive disease	7	(3.8%)	3	(3.3%)	4	(4.4%)	
OR: complete + partial response	161	(88.5%)	79	(86.8%)	82	(90.1%)	0.644^B^

^A^Fisher's Exact test for complete response vs. others; ^B^Fisher's Exact test for complete + partial response versus others

Patients undergoing B-TACE had a significantly lower retreatment rate within the first 6 months as compared to patients undergoing cTACE/DEM-TACE (9.9%% vs. 26.0%, *p* = 0.001); this significant difference was also maintained after PSM (9.9%% vs. 22.0%, *p* = 0.041) (Table 3).

**Table 3:** Number of retreatments in the total patient population before and after propensity score matching (PSM) for number of nodules, age, gender, type of TACE and Child–Pugh class

Before PSM	Total no. of patients (*n* = 525)		Non-B-TACE (*n* = 434)		B-TACE (*n* = 91)		*P*
Retreatment	122	(23.2%)	113	(26.0%)	9	(9.9%)	0.001^A^
After PSM	Total no. of patients (*n* = 182)		Non-B-TACE (*n* = 91)		B-TACE (*n* = 91)		*P*
Retreatment	29	(15.9%)	20	(22.0%)	9	(9.9%)	0.041^A^

^A^Fisher's Exact test

The type of treatment carried out under balloon-occluded TACE (B-cTACE or B-DEM-TACE) did not influence the CR rates, which were 72.7% for cTACE and 55.1% for DEM-TACE (*P* = 0.212) (Table 3-supplementary material).

In the B-TACE patients, the BOASP with the micro-balloon inflated was 64.1 ± 27.7 mmHg (min 33; max 220 mmHg) while, prior to inflation, it was 120.5 ± 36.5 mmHg; therefore, the average pressure drop was 56.4 ± 19.6 mmHg. No significant differences were observed between a BOASP value below 64 mmHg as a cut-off point, and the CR rate (67.9% vs. 58.8%, *p* = 0.749) and the OR rate (100.0% vs. 88.2%, *p* = 0.137).

The technical success rate was 100% in both study arms for selective/superselective catheterisations and in no case were the B-TACE procedures less selective than desired due to the profile of the microcatheter. No intraprocedural AEs or complications occurred in 100% of patients.

In order to avoid selection biases, the AEs were reported only in the PSM populations. The AEs were similar between the two arms, with a significant prevalence of Post-embolisation syndrome (PES) rates of (abdominal pain and nausea) in the B-TACE patients (8.8% in non-B-TACE and 41.8% in B-TACE, *p* < 0.001) (Table 4), all of whom were medically treated. All of these complications were grade 1–2 according to CTCAE version 5.

**Table 4:** Adverse events (AEs) in the propensity score matching (PSM) selected population

	PATIENT POPULATION (*n* = 182)		Non-B-TACE (*n* = 91)		B-TACE (*n* = 91)		*P*
Clinical AEs							
Post-embolisation syndrome (PES):	46	25.3%	8	8.8%	38	41.8%	P < 0.001^A^
Fever	7	3.8%	2	2.2%	5	5.5%	0.444 ^A^
Vomiting	4	2.2%	0	0.0%	4	4.4%	0.121 ^A^
Nausea	9	4.9%	0	0.0%	9	9.9%	0.003 ^A^
Abdominal pain	26	14.3%	6	6.6%	20	22.0%	0.005 ^A^
Diarrhoea	2	1.1%	2	2.2%	0	0.0%	0.497 ^A^
Fatigue	3	1.6%	3	3.3%	0	0.0%	0.246 ^A^
Biological AEs	Mean	Range	Mean	Range	Mean	Range	
Alanine aminotransferase (ALT) at baseline (IU/L)*	46.2	(10–572)	45.8	(15–435)	46.5	(10–572)	0.953^B^
aspartate aminotransferase (AST) at baseline (IU/L)*	54.6	(16–376)	52.1	(18–376)	57.0	(16–353)	0.544 ^B^
Alanine aminotransferase (ALT) at the 1-month follow-up (IU/L)	40.1	(8–145)	42.4	(10–145)	37.7	(8–130)	0.245 ^B^
aspartate aminotransferase (AST) at the 1-month follow-up (IU/L)	51.6	(11–262)	53.2	(11–234)	50.0	(14–262)	0.579 ^B^
Hyperbilirubinemia*	2	1.1%	1	1.1%	1	1.1%	1.000 ^A^
Radiological AEs							
Liver Abscess	2	1.1%	0	0.0%	2	2.2%	0.497 ^A^
Intrahepatic Arterial Pseudoaneurysm	2	1.1%	1	1.1%	1	1.1%	1.000 ^A^

Biological AEs	Mean	Range	Mean	Range	Mean	Range	
Alanine aminotransferase (ALT) at baseline (IU/L)*	46.2	(10–572)	45.8	(15–435)	46.5	(10–572)	0.953^B^
aspartate aminotransferase (AST) at baseline (IU/L)*	54.6	(16–376)	52.1	(18–376)	57.0	(16–353)	0.544 ^B^
Alanine aminotransferase (ALT) at the 1-month follow-up (IU/L)	40.1	(8–145)	42.4	(10–145)	37.7	(8–130)	0.245 ^B^
aspartate aminotransferase (AST) at the 1-month follow-up (IU/L)	51.6	(11–262)	53.2	(11–234)	50.0	(14–262)	0.579 ^B^
Hyperbilirubinemia*	2	1.1%	1	1.1%	1	1.1%	1.000 ^A^

Radiological AEs							
Liver Abscess	2	1.1%	0	0.0%	2	2.2%	0.497 ^A^
Intrahepatic Arterial Pseudoaneurysm	2	1.1%	1	1.1%	1	1.1%	1.000 ^A^

^A^Fisher's Exact test; ^B^Student’s t test. *during the first week after the procedure

In terms of radiological complications at 1 month, CT showed the development of two asymptomatic abscesses (2/91 procedures evaluated: 2.2%) in the B-TACE arm and two hepatic pseudoaneurysms (2/182: 1.1%), equally distributed in the B-TACE arm and one in the non-B-TACE arm.

### Discussion

In this multicentric study, comparing B-TACE and non-B-TACE using PSM, it has been demonstrated a clear superiority of the CR rates of B-TACE over those of non-B-TACE performed with either a conventional lipiodol-based regimen or with DEM-TACE, resulting in lower rates of retreatment needed. The primary endpoint was to compare response rates, both the OR (complete and partial) and the CR, and early retreatment rates after B-TACE versus the standard non-B-TACE procedures, using PSM to avoid biases. The ORs after B-TACE and non-B-TACE were similar after PSM; however, the CR was significantly higher for B-TACE.

The OR rates achieved after TACE are of paramount importance, since tumour response measured by the mRECIST criteria have been shown to correlate with survival outcomes in both single studies [33, 34] and in a literature-based meta-analysis [35]. A systematic review carried out in 2016, analysing 101 studies published between 1980 and 2013 (10,108 patients) to assess cTACE efficacy, established that Lipiodol-based TACE leads to an OR in 52.5% of cases [36] while, in more recent series, it increases up to nearly 100% [36, 37].

Concerning the non-B-TACE arm objective responses, the OR results of 86.8% using PSM in the present study are similar those reported after DEM-TACE by De Baere et al. [38] in 97 patients (OR: 81%), by Aliberti et al. [39] in 421 patients and by Veloso Gomes et al. [40] in 302 patients after small-bead DEM TACE (OR 94.5% at 3 months and OR 85.5% at 1 month, respectively). The present results are far higher than those of Casadaban et al. [41] in 188 patients treated with cTACE (OR 66%) and of Roth et al. [42] in 90 patients after cTACE (OR 76.7% with doxorubicin and 73.3% with idarubicin), and are better than Richter et al. [43] in the Miracle DEM-TACE study in 25 patients (OR 67%), and Guiu et al. with Idarubicin-loaded DEM-TACE with 46 study participants having an OR of 68% [44] and 72 patients having an OR of 65% [45].

In the non-B-TACE arm, the CR results of 41.8% in the present study are similar to those of the most recent series which reported CR rates ranging from 45 to 68% after 1- and 3-month cTACE [2, 37, 41] and of 48% [43], 41–40.0% [42] and 63.2% [40] after DEM-TACE.

In the B-TACE arm, the OR results of 90.1% in the present study were in line with the best responses in the literature and mirror those of three recent small series of Lucatelli et al. [33], Goldman et al. [46] and Bucalau et al. [47] regarding B-TACE. The first, after B-DEM-TACE in a series of 22 patients, reported at 1 and 3–6 months an OR rate of 90.9%-76.5% [48]; the second after B-DEM-TACE in 26 patients reported an OR rate of 93.3% after B-TACE [46], both of whose results were slightly higher than those of Bucalau et al. [47] who reported in a prospective study of B-DEM-TACE on 24 patients a one-month OR rate of 74.3%. These figures were better than those preliminarily reported by other authors [29, 49–51] after B-TACE using a miriplatin-lipiodol mixture (ORs of 63.6%, 59.6%, 57.1% and 56.3%).

In the present study, CR was specifically investigated since significantly longer overall survival (OS) has been demonstrated for patients showing a CR to the initial TACE procedure [52]. This has been called “curative” TACE by the recent Asian-Pacific consensus statement [10] in order to emphasize the difference as compared with TACE achieving a partial response in which, conversely, surviving hypoxic tumours frequently change to sarcomatous or mixed hepato-cholangiocellular phenotypes and induce vascular endothelial growth factor, which additionally promotes tumour progression [53–55].

The data in the literature comparing B-TACE to non-B-TACE CR results are scarce; they are also confounded due to different chemotherapeutics and being based on small series. A recent review of the B-TACE literature from 2014 to 2018 [49–51] observed a wide variability in CRs, ranging from low values of 8.6% with the use of miriplatin, which is known as being less effective than epirubicin [56], increasing up to 89.3% in the only study regarding B-TACE with epirubicin [57]. With B-cTACE using Miriplatin, Ogawa et al. reached a CR of 49.2% as compared with 27% for cTACE [58]; in the Bucalau [47] cohort with B-DEM-TACE, a CR of 48.7% was reached. In the B-TACE arm in the present study, the CR rate of 59.3% was superior and was parallel to the two recent retrospective studies of Lucatelli and Goldman [46, 48]. The first, after B-DEM-TACE, reported a CR rate of 41.7–52.9% at 1 and 3–6 months, and the second reported a CR rate of 60% after B-TACE, performed with either a Lipiodol-based regimen or with DEM-TACE; both were higher than those of Bucalau [47] who reported a CR rate of 41.2% in a prospective study of B-DEM-TACE involving 24 patients.

A notable reduction in the BOASP, equal to or less than 64 mmHg, has previously been demonstrated to allow higher drug deposition in targeted tumours and zone of lower resistance. Such conditions during B-TACE should provide higher rates of portal vein opacification, significantly improving cancer nodule control when compared with cTACE [24, 26]. When the BOASP after balloon inflation remains > 64 mmHg, the rate of tumour vascularity by the arterial collateral circulation (“isolated arteries”) should be evaluated [24]. In the present study, the correlation between the BOASP and CR was not statistically significant; however, a trend toward a higher OR (100.0% vs. 88.2%) was observed for lower BOASP values.

In addition, it was demonstrated that Lipiodol-based B-TACE and B-TACE with Microspheres had similar response rates, although a trend towards higher CR rates was observed for Lipiodol-based B-TACE. The similarity in efficacy of cTACE and DEM-TACE is in agreement with all previous trials [1, 2], and the non-superiority of DEM-TACE over cTACE for both tumour response and survival has been confirmed in a recent meta-analysis [3].

The desired selectivity of the treatment was not affected in any case by the use of a balloon catheter, and the technical success rate of superselective B-TACE and non-B-TACE was the same in all cases since the tip of the micro-balloon catheter (Occlusafe®) was 1.9-F, thinner than the usual microcatheters (Terumo Progreat® and Renegade™ Hi-Flo™) which have a 2.7–2.8-F tip.

The safety of the procedure was also satisfactory, with no intraprocedural complications in 100% of patients. Severe adverse events were rare and were similar between the B-TACE and the non-B-TACE arms, with a significant prevalence of medically controlled pain and nausea in the B-TACE patients. This was probably due to drug infusion and absorption both in the tumour and in the peritumoral area which could have contributed to the higher CR rates achieved, as has already been demonstrated [26], with broader necrotic areas also including satellite lesions. However, in the B-TACE arm, as compared with the incidence reported in the existing literature regarding B-TACE performed with miriplatin [28, 59], each aspect of the PES had a lower incidence. In particular, in the present B-TACE series, 5.5% experienced fever versus the reported rates of 78.4% by Ishikawa [59] and 68% by Maruyama [28], and 9.9% experienced nausea versus 28% of Maruyama’s series [28], but with the rate of abdominal pain doubled as compared with those reported by Maruyama (28% vs. 14%) [28]. Liver abscess was reported in 6% of cases in Maruyama’s series whereas its rate was far lower in the present study (2.2%); all cases were observed in the B-TACE arm, and could have been related to the more robust necrotic effect achieved by complete occlusion of the peribiliary plexus (PBP), as has already been reported [28].

The micro-balloon catheters used in B-TACE are innovative and sophisticated but costly when added to the cost of cTACE or DEM-TACE; such overcost can be justified if it reduces retreatment. In the literature, an average number of 1.8 (1.3–2.2) retreatments for cTACE, and of 2.0 (1.5–2.4) for DEM-TACE has previously been reported, with approximately 40–46% of patients retreated [2] due to an initial partial response or to recurrence as described in 27%, 42% and 65% at 6,12 and 24 months, respectively [60].

The present study points out that patients who received B-TACE had a significantly lower retreatment rate within the first 6 months as compared to patients receiving cTACE/DEM-TACE; this significant difference was also maintained after PSM. The high rate of complete response after B-TACE and, hence, the reduced need for retreatment has also recently been suggested as an alternative rescue therapy for HCC refractory to repeated cTACE [52]. In a retrospective analysis [52] of B-TACE treatment of residual or recurrent HCC after cTACE, B-TACE had a 100% OR according to the mRECIST criteria (a 75% CR and a 25% PR); time to progression was significantly longer as compared to that of the last cTACE (median 4.4 vs. 2.7 months).

The present study has some limitations. The first is related to the retrospective analysis of the data collection which included a wide range of tumour sizes; however, PSM attempted to overcome this limitation. The second may derive from the inclusion of both cTACE and DEM-TACE; however, the Authors felt that an increase in the study population compensated for this since equivalent results of both methods had, for the most part, been demonstrated. Another limitation could be related to selection bias since the patients were selected to receive B-TACE or non-B-TACE during routine clinical practice, resulting in a variability of tumour burden and results; however, PSM compensated for this limitation. Nevertheless, the inclusion of all patients eligible for TACE in this study and the liberal assessment protocols gave a realistic representation of current TACE practice.

To confirm these promising retrospective matched cohort results, future multicentric randomised controlled trials are warranted, focusing on specific and clinically relevant outcomes, and eventually being stratified for tumour size in order to better refine the patient selection criteria for B-TACE.

### Conclusion

B-TACE is safe and effective, achieving higher CR rates for treating HCCs when compared to non-B-TACE (either cTACE or DEM-TACE) which perform similarly under balloon occlusion. Patients undergoing B-TACE have a significantly lower retreatment rate within the first 6 months, but higher PES rates. A higher CR rate allows for better tumour control and possible prolonged survival.

## Supplementary Information

Below is the link to the electronic supplementary material.

Supplementary file1 (DOCX 40 KB)
