# Supplementary material for: Retrospective European Multicentric Evaluation of Selective Transarterial Chemoembolisation with and without Balloon-Occlusion in Patients with Hepatocellular Carcinoma: A Propensity Score Matched Analysis
Source: Cardiovasc Intervent Radiol. 2021 Mar 11;44(7):1048–59. doi: 10.1007/s00270-021-02805-5 (PMC8189964; doi:10.1007/s00270-021-02805-5)
Supplement: Supplementary file 1 — Supplementary file1 (DOCX 40 KB) [file 270_2021_2805_MOESM1_ESM.docx]

**SUPPLEMENTARY TABLES**

Table 1: B-TACE population: patients enrolled from the different European centres SUPPLEMENTARY 1

| Hospital | Patients treated | | Patients with follow-up | |
| --- | --- | --- | --- | --- |
| *IRCCS Azienda Ospedaliero Universitaria di Bologna, Italy | 10 | (10.4%) | 10 | (11.0%) |
| *Gustave Roussy Cancer Center, France | 10 | (10.4%) | 9 | (9.9%) |
| Sapienza University of Rome, Italy | 28 | (29.2%) | 28 | (30.8%) |
| Erasme Hospital Brussels, Belgium | 26 | (27.1%) | 26 | (28.6%) |
| IRCCS De Bellis, Castellana Grotte, Bari, Italy | 20 | (20.8%) | 18 | (19.8%) |
| Hôpital Croix Rousse Lyon, France | 2 | (2.1%) | 0 | (0.0%) |
| Overall | 96 | | 91 | |

*coordinating sites

Table 2: Baseline characteristics of the B-TACE population treated with either cTACE or DEM-TACE -

|  | B-TACE patients  (n=91) | | B-cTACE  (n=22) | | B-DEM-TACE  (n=69) | | *P* |
| --- | --- | --- | --- | --- | --- | --- | --- |
| Gender |  |  |  |  |  |  |  |
| - male | 75 | (82.4%) | 18 | (81.8%) | 57 | (82.6%) | 1.000^A^ |
| - female | 16 | (17.6%) | 4 | (18.2%) | 12 | (17.4%) |  |
| Age, mean (range) | 68.59 | (40-91) | 71.05 | (44-88) | 67.81 | (40-91) | 0.267^B^ |
| No. of nodules, mean (range) | 1.97 | (1-9) | 2.05 | (1-9) | 1.94 | (1-7) | 0.788^B^ |
| Child-Pugh class at first TACE |  |  |  |  |  |  |  |
| - A | 67 | (73.6%) | 18 | (81.8%) | 49 | (71.0%) |  |
| - B | 24 | (26.4%) | 4 | (18.2%) | 20 | (29.0%) | 0.411^A^ |
| - C | 0 | (0.0%) | 0 | (0.0%) | 0 | (0.0%) |  |

^A^ Fisher's Exact test; ^B^ Student’s t test;

Table 3: Comparison of responses between the two types of B-TACE treatment (B-cTACE vs. B-DEM-TACE).

|  | B-TACE patients  (n=91) | | Balloon occluded cTACE  (n=22) | | Balloon-occluded  DEM-TACE  (n=69) | | *P* |
| --- | --- | --- | --- | --- | --- | --- | --- |
| Best Overall Response (OR) |  |  |  |  |  |  |  |
| - complete response | 54 | (59.3%) | 16 | (72.7%) | 38 | (55.1%) | 0.212^A^ |
| - partial response | 28 | (30.8%) | 5 | (27.7%) | 23 | (33.3%) |  |
| - stable disease | 5 | (5.5%) | 0 | (0.0%) | 5 | (7.2%) |  |
| - progressive disease | 4 | (4.4%) | 1 | (4.5%) | 3 | (4.3%) |  |
| -OR: complete+partial response | 61 | (88.4%) | 21 | (95.5%) | 61 | (88.4%) | 0.446^B^ |

^A^ Fisher's Exact test for complete response vs. others; ^B^ Fisher's Exact test for complete+partial response vs. others
